# ^1^H, ^15^N and ^13^C backbone resonance assignments of the P146A variant of β-phosphoglucomutase from *Lactococcus lactis* in its substrate-free form

**DOI:** 10.1007/s12104-019-09904-y

**Published:** 2019-08-08

**Authors:** F. Aaron Cruz-Navarrete, Nicola J. Baxter, Henry P. Wood, Andrea M. Hounslow, Jonathan P. Waltho

**Affiliations:** 1 Department of Molecular Biology and Biotechnology, Krebs Institute for Biomolecular Research, The University of Sheffield, Firth Court, Western Bank, Sheffield, S10 2TN UK; 2 Manchester Institute of Biotechnology and School of Chemistry, The University of Manchester, 131 Princess Street, Manchester, M1 7DN UK

**Keywords:** Phosphoryl transfer enzyme, Backbone resonance assignment, Transverse relaxation optimised spectroscopy, General acid–base catalysis, Triple-labelled protein

## Abstract

β-Phosphoglucomutase (βPGM) is a magnesium-dependent phosphoryl transfer enzyme that catalyses the reversible isomerisation of β-glucose 1-phosphate and glucose 6-phosphate, via two phosphoryl transfer steps and a β-glucose 1,6-bisphosphate intermediate. Substrate-free βPGM is an essential component of the catalytic cycle and an understanding of its dynamics would present significant insights into βPGM functionality, and enzyme catalysed phosphoryl transfer in general. Previously, 30 residues around the active site of substrate-free βPGM_WT_ were identified as undergoing extensive millisecond dynamics and were unassignable. Here we report ^1^H, ^15^N and ^13^C backbone resonance assignments of the P146A variant (βPGM_P146A_) in its substrate-free form, where the K145–A146 peptide bond adopts a *trans* conformation in contrast to all crystal structures of βPGM_WT_, where the K145–P146 peptide bond is *cis.* In βPGM_P146A_ millisecond dynamics are suppressed for all but 17 residues, allowing 92% of backbone resonances to be assigned. Secondary structure predictions using TALOS-N reflect βPGM crystal structures, and a chemical shift comparison between substrate-free βPGM_P146A_ and βPGM_WT_ confirms that the solution conformations are very similar, except for the D137–A147 loop. Hence, the isomerisation state of the 145–146 peptide bond has little effect on structure but the *cis* conformation triggers millisecond dynamics in the hinge (V12–T16), the nucleophile (D8) and residues that coordinate the transferring phosphate group (D8 and S114–S116), and the D137–A147 loop (V141–A142 and K145). These millisecond dynamics occur in addition to those for residues involved in coordinating the catalytic Mg^II^ ion and the L44–L53 loop responsible for substrate discrimination.

## Biological context

β-Phosphoglucomutase (βPGM, EC 5.4.2.6) from *Lactococcus lactis* is a magnesium-dependent phosphoryl transfer enzyme of the haloacid dehalogenase superfamily (Lahiri et al. 2002a; Allen and Dunaway-Mariano 2004; Dai et al. 2009). In the catabolism of maltose and trehalose, βPGM catalyses the reversible isomerisation of β-glucose 1-phosphate (βG1P) and glucose 6-phosphate (G6P). During catalysis, βG1P binds to phosphorylated βPGM (βPGM^P^, phosphorylated on residue D8) forming β-glucose 1,6-bisphosphate (βG16BP), which is released to solution. Subsequent rebinding of βG16BP in the alternate orientation to non-phosphorylated, substrate-free βPGM results in dephosphorylation of βG16BP, with the formation of G6P (which enters glycolysis) and the regeneration of βPGM^P^ (Zhang et al. 2005; Dai et al. 2006). The βPGM gene (*pgmB*) is located on the trehalose operon and is induced by maltose or trehalose in the growth medium but is repressed by the presence of glucose or lactose (Qian et al. 1994, 1997). A βPGM knockout mutant strain of *L. lactis* shows impaired growth when maltose is used as the only carbon source, coupled with an intracellular accumulation of trehalose 6-phosphate and polysaccharide molecules composed of α-1,4-linked glucose units (Levander et al. 2001). Such a perturbation of the metabolic flux highlights the crucial role that βPGM plays in mediating the efficient utilisation of carbohydrate species in *L. lactis* metabolism.

Wild-type βPGM (βPGM_WT_) together with a series of variants have been studied extensively using kinetic experiments (Zhang et al. 2005; Dai et al. 2006, 2009; Goličnik et al. 2009), X-ray crystallography (Lahiri et al. 2002a, b, 2003; Tremblay et al. 2005; Baxter et al. 2010; Griffin et al. 2012; Jin et al. 2014; Johnson et al. 2018), NMR spectroscopy (Baxter et al. 2006, 2008, 2009, 2010; Griffin et al. 2012; Jin et al. 2014; Johnson et al. 2018) and density functional theory approaches (Webster 2004; Marcos et al. 2010, Elsässer et al. 2012; Barrozo et al. 2018) and it is considered as an archetypal system for enzyme catalysed phosphoryl transfer reactions. Structural analysis coupled with metal-fluoride ground state and transition state analogue (TSA) complexes have allowed the atomic resolution description of several discrete species found in the catalytic cycle i.e. substrate-free βPGM_WT_ (PDB: 2WHE; Baxter et al. 2010), a ground state βPGM_WT_^P^ analogue (βPGM_WT_:BeF_3_ complex; PDB: 2WFA; Griffin et al. 2012), two ground state βPGM_WT_^P^:G6P complexes (βPGM_WT_:BeF_3_:G6P complexes; PDB: 2WF8; PDB: 2WF9; Griffin et al. 2012), two βPGM_D10N_:βG16BP complexes (PDB: 5OK1; PDB: 5OK0; Johnson et al. 2018), a βPGM_WT_^P^:G6P TSA complex (βPGM_WT_:MgF_3_:G6P TSA complex; PDB: 2WF5; Baxter et al. 2010) and a βPGM_WT_^P^:βG1P TSA complex (βPGM_WT_:MgF_3_:βG1CP TSA complex; PDB: 4C4R; Jin et al. 2014). The enzyme active site is located in the cleft formed between the α/β core domain (M1–D15 and S88–K216) and the α-helical cap domain (T16–V87). During catalysis, domain reorientation through hinge residue (D15–T16 and V87–S88) rearrangement results in closure and opening of the active site cleft facilitating substrate binding and product release. Two phosphate group binding sites are present, one in a *proximal* site adjacent to the carboxylate nucleophile (residue D8) (Lahiri et al. 2002a) and the catalytic Mg^II^ ion (Lahiri et al. 2002a), and the other in a *distal* site located ~ 8 Å away in the closed enzyme (Lahiri et al. 2003). The carboxylate group of the assigned general acid–base (residue D10) (Dai et al. 2009) populates two orientations depending on the degree of active site closure. In the structures of substrate-free βPGM_WT_ and the βPGM_WT_^P^ analogue, the active site cleft is open and the D10 carboxylate group is not engaged in the active site, whereas in the closed βPGM_WT_^P^:G6P, βPGM_D10N_:βG16BP, βPGM_WT_^P^:G6P TSA and βPGM_WT_^P^:βG1P TSA complexes, the carboxylate group is positioned to facilitate general acid–base catalysis promoting phosphoryl transfer (Johnson et al. 2018). Key roles for several residue segments in the active site have been identified including, coordination of the transferring phosphate group in the *proximal* site (V9, D10, S114, A115 and K145) (Lahiri et al. 2003), coordination of the phosphate group of the substrate in the *distal* site (R49, S116, K117 and N118) (Lahiri et al. 2003), substrate discrimination and binding (L44–L53) (Lahiri et al. 2004) and coordination of the catalytic Mg^II^ ion (D10, E169 and D170) (Lahiri et al. 2002a).

Previously, the solution behaviour of substrate-free βPGM_WT_ was investigated by NMR spectroscopy and a backbone resonance assignment was determined (BMRB: 7235; Baxter et al. 2006). However, 30 residues (D8–T16, R38, L44–L53, S114–N118, V141–A142, K145 and S171–Q172) located primarily in the active site loops remained unassigned in the ^1^H–^15^N TROSY spectrum, most likely due to extensive conformational intermediate exchange dynamics occurring on the millisecond timescale, which results in broadening of the correlations beyond the limits of detection. Substrate-free βPGM is an essential component of the catalytic cycle and an understanding of the dynamics of key residue segments would present significant insights into βPGM functionality. Accordingly, a series of single site variants of βPGM was screened to establish whether any improvement in spectral quality could be obtained. Of the variants tested, the P146A variant of βPGM (βPGM_P146A_) reduced the intermediate exchange dynamics by the strongest extent and so was investigated further. On the basis of the conformational properties of alanine, βPGM_P146A_ is expected to adopt a *trans* K145–A146 peptide bond as the dominant population. In contrast, all of the reported crystal structures described for βPGM_WT_, indicate a *cis* K145–P146 peptide bond within the D137–A147 loop. Consequently the isomerisation state of the 145–146 peptide bond presents a trigger for some of the intermediate exchange dynamics observed. Preliminary kinetics experiments using methods described previously (Johnson et al. 2018) indicate that βPGM_P146A_ is active. Complete equilibration of 10 mM βG1P with G6P by 3 μM βPGM_P146A_ was achieved in 1.5 h. Here, we report the ^1^H_N_, ^15^N, ^13^C_α_, ^13^C_β_ and ^13^C’ backbone resonance assignments of substrate-free βPGM_P146A_, including the resonances of many residues that were previously unassigned in substrate-free βPGM_WT_.

## Methods and experiments

### Protein expression and purification

Site-directed mutagenesis (QuikChange II Site-Directed Mutagenesis Kit, Agilent Technologies) of the *pgmB* from *Lactococcus lactis* cloned in the pET-22b(+) expression plasmid was employed to generate βPGM_P146A_ using primers with single-site base changes. Successful mutagenesis was confirmed by DNA sequencing. The plasmid was transformed into *Escherichia coli* strain BL21(DE3) cells (Stratagene) and ^2^H,^15^N,^13^C-labelled βPGM_P146A_ (25 kDa) was expressed in defined isotopically labelled minimal media (Reed et al. 2003). The cells were grown at 37 °C with shaking until OD_600nm_ = 0.6, at which point they were cooled to 25 °C and induced with isopropyl β-d-1-thiogalactopyranoside (IPTG) to a final concentration of 0.5 mM. Cells were incubated for a further 18 h and were harvested by centrifugation at 10,000 rpm for 10 min. The cell pellet was resuspended in ice-cold standard working buffer (50 mM K^+^ HEPES pH 7.2, 5 mM MgCl_2_, 2 mM NaN_3_, 1 mM EDTA) supplemented with cOmplete™ protease inhibitor cocktail (Roche) (one tablet per 50 mL cell suspension). The cell suspension was lysed on ice using 6 cycles of sonication with pulsation for 20 s followed by 60 s cooling intervals. The cell lysate was then separated by ultracentrifugation at 20,000 rpm (Beckman Coulter Avanti centrifuge using rotor JA-20) for 35 min at 4 °C. The cleared cell lysate was filtered using a 0.22 µm syringe filter (Merck Millipore) and loaded onto a DEAE-Sepharose fast flow anion-exchange column connected to an ÄKTA purification system (GE Healthcare) that had been washed previously with 1 column volume of 6 M guanidine chloride, 1 column volume of 1 M NaOH and equilibrated with 5 column volumes of standard working buffer. Proteins bound to the DEAE-Sepharose column were eluted with a gradient of 0 to 50% standard working buffer containing 1 M NaCl. Fractions were checked for the presence of βPGM_P146A_ by SDS-PAGE, pooled together and concentrated by Vivaspin (10 kDa MWCO, Sartorius). The protein sample was loaded onto a prepacked Hiload 26/60 Superdex 75 size-exclusion column connected to an ÄKTA purification system previously washed with 1 column volume of 1 M NaOH and equilibrated with 1.5 column volumes of standard working buffer containing 1 M NaCl. Fractions containing βPGM_P146A_ were checked for purity by SDS-PAGE, pooled together and buffer exchanged into standard working buffer and concentrated to ~ 1.6 mM by Vivaspin (10 kDa MWCO) for storage as 1 mL aliquots at − 20 °C. No procedure was necessary to promote back exchange to amide protium atoms in perdeuterated βPGM_P146A_. Protein concentrations were estimated by absorbance at 280 nm (ε_280_ = 19940 M^−1^ cm^−1^). All reagents were of analytical grade and were purchased from Sigma-Aldrich (UK), except for the stable isotopically-labelled compounds ^15^NH_4_Cl (99%), ^13^C, ^2^H_7_-d-Glucose (U–^13^C_6_, 99%; 1,2,3,4,5,6,6-d_7_ 97–98%) and ^2^H_2_O (99.8%), which were purchased from CortecNet (France) and used as received.

### NMR experiments

The NMR experiments were acquired using samples loaded into 5-mm NMR tubes, which contained 1.2 mM ^2^H,^15^N,^13^C-labelled βPGM_P146A_ in standard working buffer supplemented with ^2^H_2_O (10% v/v) for the deuterium lock and 1 mM trimethylsilyl propanoic acid (TSP) as a chemical shift reference. All experiments were recorded at 298 K using an 800 MHz Bruker Avance I spectrometer fitted with a 5-mm TXI probe equipped with z-axis gradients and running TopSpin software version 2.1. For the backbone ^1^H, ^15^N, ^13^C resonance assignment of substrate-free βPGM_P146A_, 2D ^1^H–^15^N TROSY and TROSY-based 3D HNCA, HN(CO)CA, HNCACB, HN(CO)CACB, HN(CA)CO and HNCO spectra were acquired using standard Bruker pulse sequences. ^1^H chemical shifts were referenced relative to the internal TSP signal resonating at 0.0 ppm, whereas ^15^N and ^13^C chemical shifts were referenced indirectly using nuclei-specific gyromagnetic ratios.

## Resonance assignments and data deposition

**Fig. 1:**
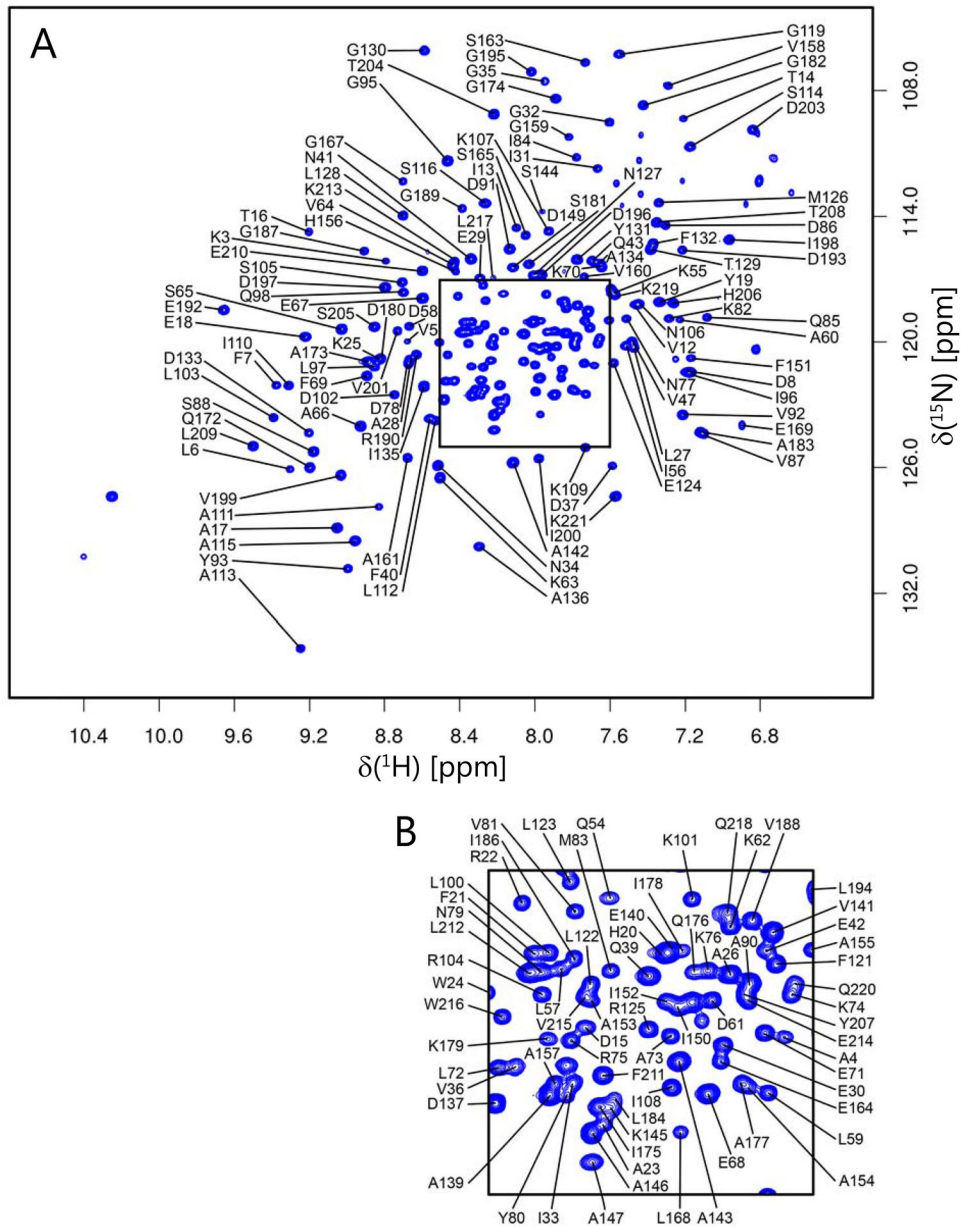
^1^H–^15^N TROSY spectrum of ^2^H,^15^N,^13^C-labelled substrate-free βPGM_P146A_ in 50 mM K^+^ HEPES pH 7.2, 5 mM MgCl_2_, 2 mM NaN_3_, 1 mM EDTA, 10% v/v ^2^H_2_O and 1 mM TSP recorded on an 800 MHz spectrometer at 298 K. **a** The full spectrum is shown together with **b** an expansion of the crowded region. The assignments of the backbone amide resonances are indicated by residue type and sequence number

Backbone ^1^H_N_, ^15^N, ^13^C_α_, ^13^C_β_ and ^13^C’ chemical shifts were assigned for substrate-free βPGM_P146A_ using standard triple resonance methodology (Gardner and Kay 1998). The processing of spectra and peak picking were performed using FELIX (Felix NMR, Inc.). Frequency matching of the backbone assignments was achieved using a simulated annealing algorithm employed by the “asstools” assignment program (Reed et al. 2003). The backbone ^1^H_N_, ^15^N, ^13^C_α_, ^13^C_β_ and ^13^C’ chemical shifts have been deposited in the BioMagResBank (http://www.bmrb.wisc.edu/) under the BMRB accession code 27920. Excluding the nine proline residues and the N-terminal methionine residue, 194 out of a possible 211 residues were assigned in the ^1^H–^15^N TROSY spectrum (Fig. 1). In total, 93.4% of all backbone resonances were assigned (91.9% of ^1^H_N_, 91.9% of ^15^N, 94.6% of ^13^C_α_, 93.7% of ^13^C_β_ and 94.6% of ^13^C′ nuclei).

**Fig. 2:**
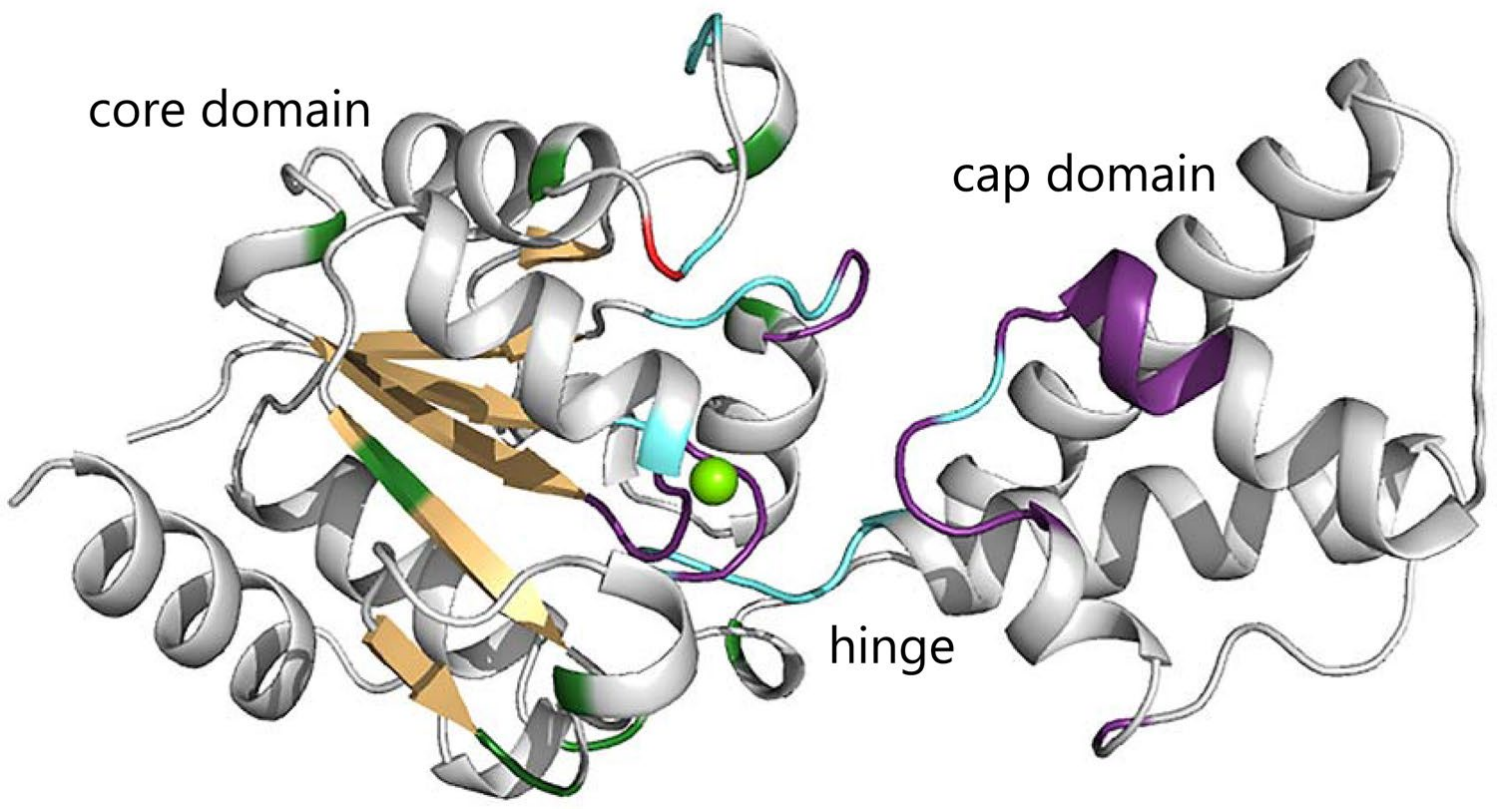
Cartoon representation of the substrate-free βPGM_WT_ crystal structure (PDB: 2WHE) highlighting the extent of backbone amide resonance assignments for substrate-free βPGM_P146A_ and substrate-free βPGM_WT_. Assigned residues for βPGM_P146A_ are coloured white (for loops and α-helices) and tan (for β-strands), with proline residues coloured green and residues that were unassigned in βPGM_WT_ coloured cyan. Unassigned residues for βPGM_P146A_ (L9, D10, G11, R38, L44, K45, G46, S48, R49, E50, D51, S52, L53, K117, N118, D170 and S171) are coloured purple and the 30 unassigned residues for βPGM_WT_ are listed here for comparison (D8, L9, D10, G11, V12, I13, T14, D15, T16, R38, L44, K45, G46, V47, S48, R49, E50, D51, S52, L53, S114, A115, S116, K117, N118, V141, A142, K145, S171 and Q172). The location of the P146A mutation site is highlighted with a red backbone and the catalytic magnesium ion is shown as a green sphere

There are 17 residues that remain unassigned in the ^1^H–^15^N TROSY spectrum of substrate-free βPGM_P146A_ (L9, D10, G11, R38, L44, K45, G46, S48, R49, E50, D51, S52, L53, K117, N118, D170 and S171) compared with 30 residues in substrate-free βPGM_WT_ (Fig. 2). From the crystal structures of substrate-free βPGM_WT_ (PDB: 2WHE) and the βPGM_WT_:MgF_3_:G6P TSA complex (PDB: 2WF5), all of the unassigned residues in substrate-free βPGM_P146A_ (except for R38) are situated within the active site and have significant roles in the catalytic cycle of the enzyme. Residues L9, D10 and G11 are key components of the *proximal* site, with D8 identified as the nucleophile, D10 assigned as the general acid–base and residues D10, D170 and S171 comprising the catalytic Mg^II^ ion binding site. Residues R49, K117 and N118 coordinate the phosphate group of the substrate in the *distal* binding site and the active site loop L44–L53 in the cap domain comprises part of a helix–loop–helix motif and is involved in substrate discrimination and binding. Due to the involvement of these residue segments in the catalytic cycle, it is likely that conformational exchange dynamics between two (or more) similarly populated forms is still occurring on the millisecond timescale in substrate-free βPGM_P146A_, resulting in the attenuation of ^1^H–^15^N TROSY correlations beyond the limits of detection. Increased solvent exposure of the amide group of R38 through perturbation of hydrogen bonding with neighbouring sidechain groups may be coupled with helix-fraying exchange behaviour within the second α-helix (D37–E42) and the first turn of the third α-helix (S48–D58) of the cap domain, resulting in a loss of the ^1^H–^15^N TROSY correlation.

**Fig. 3:**
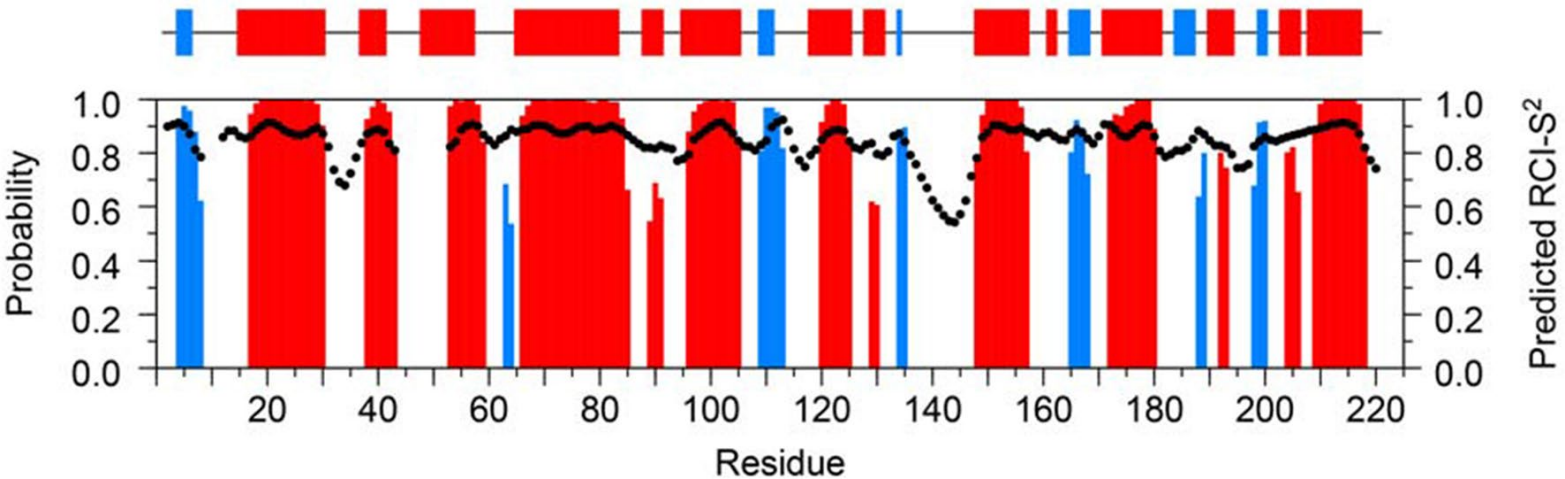
Predicted secondary structure content and residue-specific random coil index order parameters (RCI-S^2^) of substrate-free βPGM_P146A_ obtained with the TALOS-N webserver (Shen and Bax 2013) using the backbone ^1^H_N_, ^15^N, ^13^C_α_, ^13^C_β_ and ^13^C′ chemical shifts. The secondary structure prediction is shown as red bars for α-helices and blue bars for β-strands, with the height of the bars representing the probability assigned by the software. As a comparison, the secondary structure observed in the substrate-free βPGM_WT_ crystal (PDB: 2WHE) is shown at the top of the figure in the same colour representation. The predicted RCI-S^2^ values are shown as black circles

The secondary structure content and residue-specific random coil index order parameters (RCI-S^2^) of substrate-free βPGM_P146A_ were predicted by uploading the backbone ^1^H_N_, ^15^N, ^13^C_α_, ^13^C_β_ and ^13^C’ chemical shifts to the TALOS-N webserver (Shen and Bax 2013). The predicted secondary structure for the solution conformation of substrate-free βPGM_P146A_ compares well with the secondary structure present in the substrate-free βPGM_WT_ crystal (PDB: 2WHE) (Fig. 3). In addition, residues with the highest values of RCI-S^2^ are located in well-defined secondary structure elements, whereas residues with the lowest values (having more random coil-like chemical shifts) correspond to loop regions in the crystal structure. Together, these data are in very good agreement, which indicates that the solution conformation is similar to the protein structure observed in the substrate-free βPGM_WT_ crystal and provides confidence in the assignments of substrate-free βPGM_P146A_.

**Fig. 4:**
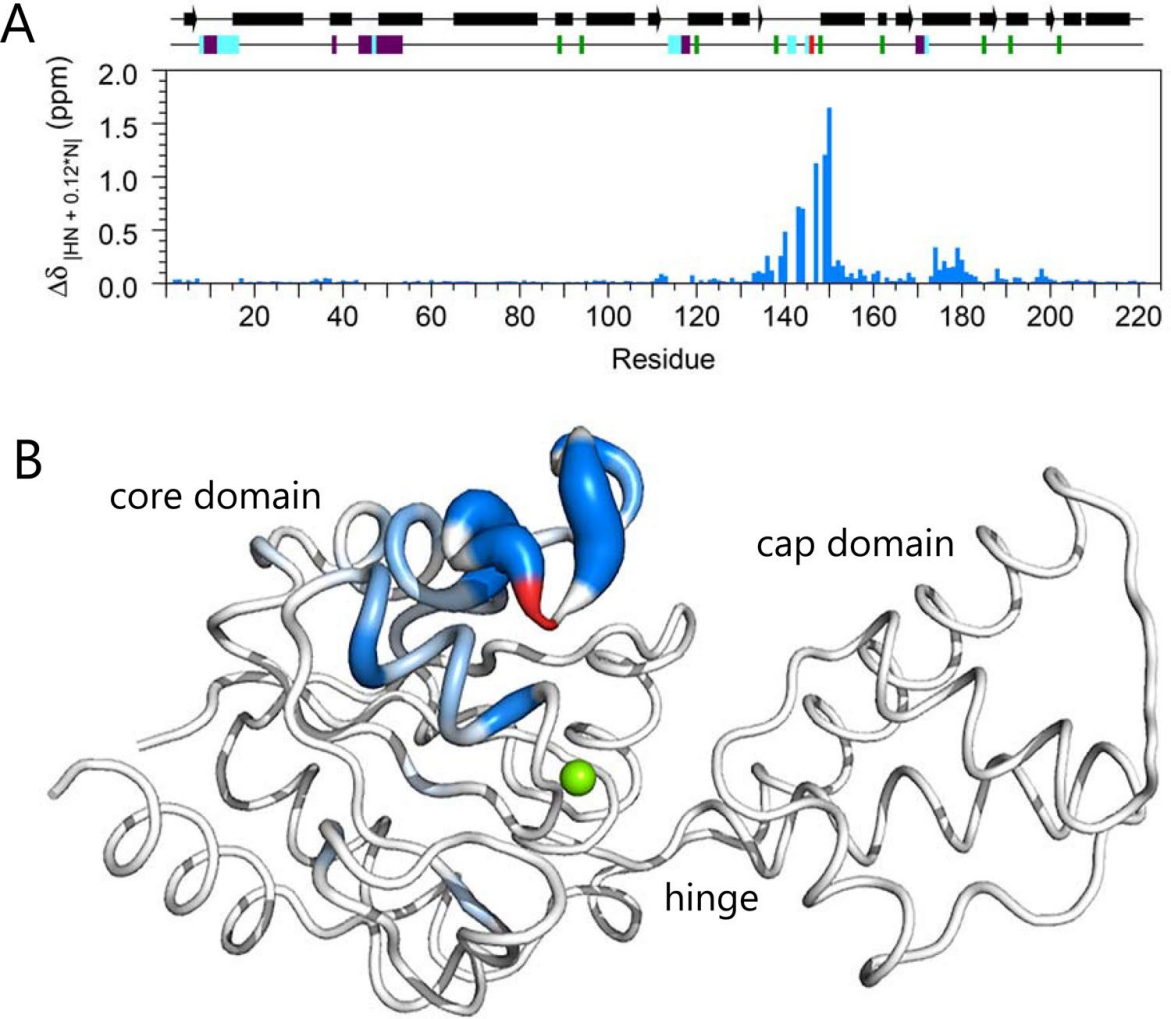
A chemical shift comparison between substrate-free βPGM_P146A_ and substrate-free βPGM_WT_. **a** Histogram of residue-specific chemical shift changes calculated between βPGM_P146A_ and βPGM_WT_ (BMRB: 7235; Baxter et al. 2006) as Δδ_|HN + 0.12*N|_ = [Δδ_HN_^2^ + (0.12 × Δδ_N_)^2^]^1/2^, where Δδ_X_ = δ_X:βPGM:P146A_ − δ_X:βPGM:WT_ and X = ^1^H_N_ or ^15^N nuclei of the backbone amide group. Secondary structure elements from substrate-free βPGM_WT_ (PDB: 2WHE) are indicated as bars for α-helices and arrows for β-strands at the top of the panel. Proline residues, the location of the P146A mutation site and unassigned residues in the ^1^H–^15^N TROSY spectra of βPGM_P146A_ and βPGM_WT_ are shown as green, red, purple and cyan rectangles, respectively at the top of the panel. **b** Structure of substrate-free βPGM_WT_ (PDB: 2WHE) with residues coloured according to Δδ_|HN + 0.12*N|_ (Δδ_|HN + 0.12*N|_ > 0.08 ppm) with the intensity of colour and thickness of the backbone corresponding to larger values. The location of the P146A mutation site is highlighted with a red backbone and the catalytic magnesium ion is shown as a green sphere

A chemical shift comparison between substrate-free βPGM_P146A_ and substrate-free βPGM_WT_ (BMRB: 7235; Baxter et al. 2006) also supports the conclusion that the solution conformations of the two proteins are very similar. Negligible Δδ values are observed for all residues of the cap domain and small Δδ values are noted for the majority of residues present in the core domain (Fig. 4). However, some larger Δδ values (0.08 < Δδ < 1.65 ppm) are observed primarily for two contiguous residue segments (D133–A153 and G174–S181), which are located within ~ 8 Å of the P146A mutation site. For a conservative single site amino acid substitution in a protein, chemical shift perturbations caused by changes in the local chemical environment are usually restricted to the immediate vicinity (within ~ 4 Å) of the mutation site (Baxter et al. 2017). Here, the size and the more widespread distribution of the chemical shift changes, together with a propagation of effects through several secondary structure elements, strongly suggest that the conformation of the D137–A147 loop is different in the two proteins. Conformational heterogeneity in this loop is observed when comparing crystal structures of substrate-free βPGM_WT_ (e.g. PDB: 1ZOL versus 2WHE; Zhang et al. 2005; Baxter et al. 2010). The source of this difference in structure is most probably associated with the isomerisation state of the K145–X146 peptide bond. For βPGM_WT_, all the reported crystal structures have a *cis* K145–P146 peptide bond and this isomer is also populated in solution as P146 δ^13^C_β_ = 34.8 ppm (Shen and Bax 2010). For βPGM_P146A_, a regular *trans* K145–A146 peptide bond is adopted according to TALOS-N, as expected on the basis of the conformational properties of alanine.

In conclusion, the isomerisation state of the K145–X146 peptide bond appears to lead to a difference in the active site dynamics, where the *cis* conformation triggers millisecond exchange for residues of the hinge (V12–T16), the nucleophile (D8) and residues responsible for coordinating the transferring phosphate group in the *proximal* site (D8 and S114–S116), and residues of the D137–A147 loop (V141–A142 and K145). These occur in addition to millisecond dynamics for residues involved in the coordination of the catalytic Mg^II^ ion and for the L44–L53 loop responsible for substrate discrimination and binding.
